# Ex Vivo Feedback Control of Neurotransmission Using a Photocaged Adenosine A1 Receptor Agonist

**DOI:** 10.3390/ijms23168887

**Published:** 2022-08-10

**Authors:** Erine Craey, Fabian Hulpia, Jeroen Spanoghe, Simona Manzella, Lars E. Larsen, Mathieu Sprengers, Dimitri De Bundel, Ilse Smolders, Evelien Carrette, Jean Delbeke, Kristl Vonck, Paul Boon, Serge Van Calenbergh, Wytse J. Wadman, Robrecht Raedt

**Affiliations:** 1 4Brain, Department of Head and Skin, Ghent University, 9000 Ghent, Belgium; 2 Laboratory of Medicinal Chemistry, Department of Pharmaceutics, Ghent University, 9000 Ghent, Belgium; 3 Research Group Experimental Pharmacology, Department of Pharmaceutical Chemistry, Drug Analysis and Drug Information, Center for Neurosciences, Vrije Universiteit Brussel, 1090 Brussels, Belgium

**Keywords:** adenosine A_1_ receptor, caged compounds, hippocampus, photopharmacology

## Abstract

We report the design, synthesis, and validation of the novel compound photocaged N^6^-cyclopentyladenosine (cCPA) to achieve precisely localized and timed release of the parent adenosine A1 receptor agonist CPA using 405 nm light. G_i_ protein-coupled A_1_ receptors (A_1_Rs) modulate neurotransmission via pre- and post-synaptic routes. The dynamics of the CPA-mediated effect on neurotransmission, characterized by fast activation and slow recovery, make it possible to implement a closed-loop control paradigm. The strength of neurotransmission is monitored as the amplitude of stimulus-evoked local field potentials. It is used for feedback control of light to release CPA. This system makes it possible to regulate neurotransmission to a pre-defined level in acute hippocampal brain slices incubated with 3 µM cCPA. This novel approach of closed-loop photopharmacology holds therapeutic potential for fine-tuned control of neurotransmission in diseases associated with neuronal hyperexcitability.

## 1. Introduction

The adenosine A_1_ receptor (A_1_R) is a G_i/o_-coupled receptor abundantly expressed throughout the body [1,2,3]. Receptor activation catalyzes the nucleotide exchange reaction (GDP to GTP) on the heterotrimeric G-protein (Gαβγ), which dissociates into Gα and Gβγ subunits that interact with downstream effectors. In neurons, activation of the A_1_R leads to suppression of neurotransmission. Presynaptically, voltage-gated Ca^2+^ channels are inhibited, resulting in reduced neurotransmitter release. Postsynaptically, G protein-mediated inwardly rectifying potassium channels are activated, resulting in membrane hyperpolarization [4,5,6].

The strong potential for modulation of neurotransmission makes A_1_Rs a hypothetically attractive therapeutic target for diseases associated with neuronal hyperexcitability, such as epilepsy, pain and depression [7,8]. However, targeting A_1_Rs with adenosine or more potent and selective agonists for therapeutic purposes is challenging due to their ubiquitous presence and the resulting high risk of side effects. Moreover, systemic application is hampered by the relatively slow ligand delivery, which is particularly problematic when fast intervention is needed in the targeted brain region. 

These drawbacks may be addressed by a more controlled application of the ligand. We explore the photopharmacological approach known as photocaging in which a bioactive ligand is typically linked to a photocleavable group that renders the ligand inactive to its target receptor(s). Tissue illumination with light of an appropriate wavelength then irreversibly releases the ligand with high spatiotemporal resolution and restores its activity [9,10,11]. The potential of photocaged receptor ligands as therapeutic tools has already been demonstrated in animal models for pain, movement disorders, psoriasis and epilepsy [12,13,14,15,16].

Here, we present the synthesis and validation of a coumarin-caged derivative of the selective and well-established A_1_R agonist N^6^-cyclopentyladenosine (CPA); i.e., cCPA. The selected coumarin cage is biologically safe and can be split within milliseconds with light of 405 nm [10,11,12,13,14,15]. The affinity loss upon conversion of CPA to cCPA was first validated in an isolated cell preparation. Next, we explored the applicability of cCPA in acute rat hippocampal brain slices, wherein extracellular field potential recordings allowed monitoring of neurotransmission. Based on the observed dynamics of the CPA response on neurotransmission, we investigated whether local field potentials could be used as the control variable in a closed-loop system that triggered light flashes whenever their amplitude exceeded a predefined level. Our combination of real-time field potential monitoring and photopharmacological modulation makes it possible to fine-tune the strength of neurotransmission at a predefined stable level. These results can guide novel attempts for future therapeutic interventions that require high spatiotemporal control over neuronal excitability.

## 2. Results and Discussion

### 2.1. Design and Synthesis of cCPA

**Scheme 1:**
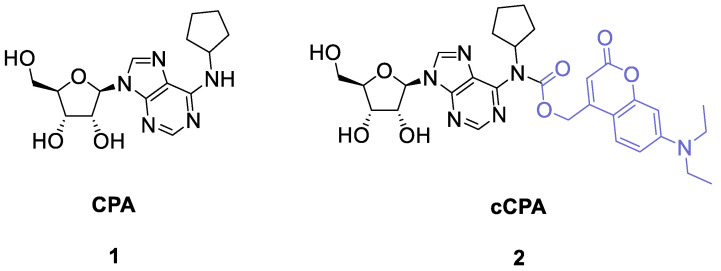
Design of cCPA.

**Scheme 2:**
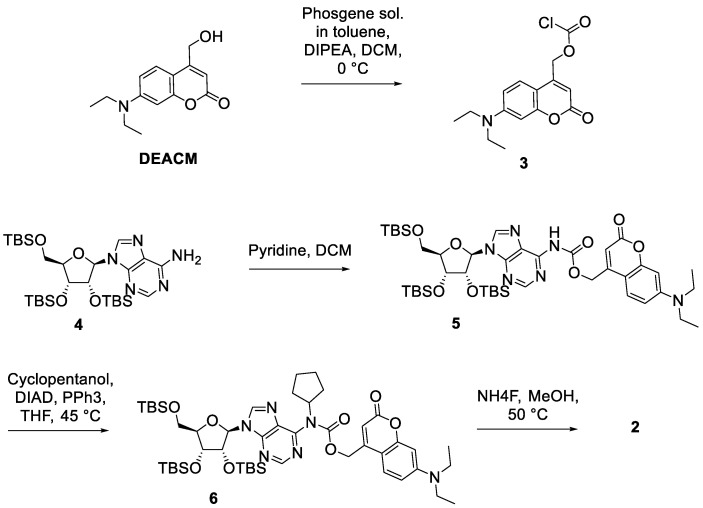
Synthesis of cCPA.

Bioactivity of parent CPA (**1**, Scheme 1) was effectively masked with the coumarin-derivative 7-(diethylamino)-4-(hydroxymethyl)-2H-chromen-2-one (DEACM) to produce cCPA (**2**, Scheme 1), which enables photolysis upon illumination with 405 nm light. The synthesis is depicted in Scheme 2 (see Materials and Methods and Supplementary Figure S1). Briefly, O-persilyl-protected adenosine (**4**) was carbamoylated [17,18] with carbonochloridate (**3**), prepared from DEACM. The resulting carbamate (**5**) was cyclopentylated under Mitsunobu conditions. Final removal of the silyl protecting groups afforded the desired cCPA (**2**). Importantly, a competitive binding experiment in HEK293 cells showed less than 20% displacement of [³H]DPCPX by 10 µM cCPA (Supplementary Figure S2), indicating that the affinity of cCPA for the human A_1_R is at least 1,000-fold weaker compared to the parent CPA (K_i_ = 2.3 nM) [19]. This demonstrates that caging effectively prevents binding to the A_1_R.

### 2.2. Light-Triggered Release of CPA

**Figure 1:**
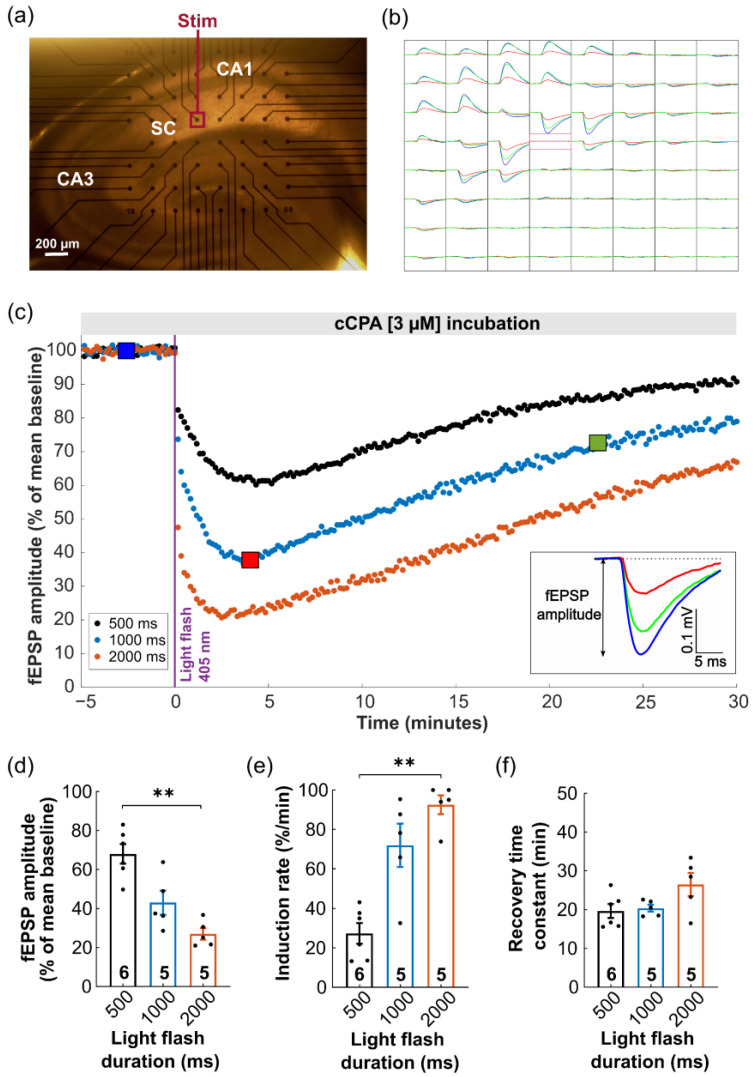
Flash photolysis releases CPA and leads to A_1_R activation. (**a**) Transverse hippocampal slice positioned on a 60 channel MEA. One electrode (Stim) in the Schaffer collaterals (SC) was selected to deliver electrical stimuli, whereas the surrounding electrodes recorded field potentials: (**b**) negative in the dendritic region and positive in the somatic region. (**c**) Time course of fEPSP amplitude in three representative slices. At zero time a light flash of variable duration was delivered to release CPA, which transiently reduced fEPSP amplitude. Inset shows fEPSP traces at three different timepoints upon a 1000 ms flash as indicated by square markers: baseline (blue), near the minimum of the response (red) and near the end of recovery (green). Identical color coding is used in (**b**), illustrating the spatial distribution of the fEPSPs. (**d**) Maximal relative reduction of fEPSP amplitude was dependent on flash duration. (**e**) Induction rate determined in the first 30 s after the flash was also related to flash duration. (**f**) The recovery time constant was similar regardless of flash duration. For (**d**–**f**), bars indicate mean ± SEM, number of slices within the bars. ** *p* < 0.01, Kruskal–Wallis test with post hoc Dunn’s multiple comparison test.

The use of cCPA for controlled modulation of neurotransmission was studied in acute rat hippocampal slices using extracellular recordings implemented with a multielectrode array (MEA) (Figure 1a). Afferent axons of CA3 pyramidal neurons (Schaffer collaterals) were electrically stimulated to evoke field potentials in the CA1 region. These responses correspond to the excitatory postsynaptic potential (fEPSP, Figure 1b) and, therefore, reflect the strength of neurotransmission. Repeated stimulation with 10 s intervals at an intensity that produced an fEPSP amplitude that was 50% of its largest response was used to continuously monitor neurotransmission strength. Hippocampal slices were incubated with 3 µM cCPA, a concentration that did not affect fEPSP amplitude without illumination (*n* = 14, Supplementary Figure S3). LED light flashes (405 nm) lasting 500, 1000 or 2000 ms were used to release CPA. On a timescale of seconds to minutes, a clear reduction of fEPSP amplitude was seen to respectively 68 ± 5%, 43 ± 6% and 27 ± 3% of mean baseline value (Figure 1c,d). Similar reductions in fEPSP amplitude have been reported after incubation of hippocampal slices in nanomolar concentrations of CPA [20,21,22]. The fEPSP slowly recovered towards baseline levels in tens of minutes. A linear regression of the falling slope directly after uncaging yielded induction rates for the three light flash durations of respectively 27 ± 5%/min, 72 ± 11%/min and 93 ± 5%/min (Figure 1e). The recovery of fEPSP amplitude was described with a single exponential function with a time constant that did not differ for the three flash durations (Figure 1f, mean value 22 ± 1 min). 

The processes that underlie this response span from microseconds to minutes and are much more complicated than can be captured by a single induction rate and recovery time constant. Uncaging is very fast, but as only a small fraction of molecules absorb a photon, longer illumination results in higher CPA release. Studies evaluating the effects of the parent CPA in hippocampal slices demonstrated a 50% reduction in fEPSP amplitude at 100-fold lower concentrations than the 3 µM cCPA used in our study [20,21,22]. Thus, we estimated that at most 1% of cCPA is uncaged and that the rise in CPA concentration is roughly proportional to the light flash duration. The activated A_1_Rs lead to G_i_ protein dissociation at a rate that is related to receptor occupation [23]. The activated G_i_ protein subunits execute their physiological functions both at the presynaptic and postsynaptic levels. Recovery is also more complex than suggested by the single exponential decay, as at least three processes must be involved: (1) dissociation of CPA from the A_1_R, and, in that regard, a previous study on CHO cells reported a ligand dissociation rate of 0.24 min^−1^ [24]; (2) diffusion of CPA out of the slice and MEA chamber, which has a time constant of a few minutes; (3) termination of the physiological effects when the Gα and Gβγ subunits are inactivated, reassemble to the heterotrimeric complex and lose their physiological effect. The latter effect seems to be the slowest one and apparently represents the major determinant of the observed recovery time constant. 

### 2.3. Proof-of-Principle: Closed-Loop Photopharmacological Control of Neurotransmission

**Figure 2:**
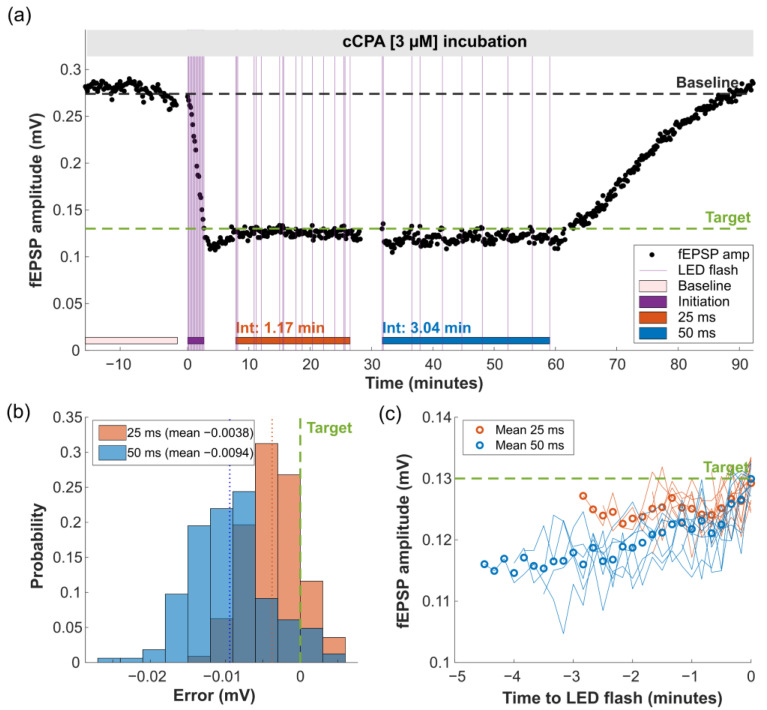
Illustration of closed-loop CPA-mediated photopharmacological control of neurotransmission. (**a**) Time course of the fEPSP amplitude in the presence of cCPA. Following baseline, the feedback loop was closed at zero time. Initially, each fEPSP triggered a flash of 25 ms. After a short undershoot, the amplitude finally settled around the target value. For the next 20 min, flashes with a mean interval of 1.17 min kept the fEPSP amplitude close to the target. When the flash duration was doubled to 50 ms, the mean flash interval increased to 3.04 min. (**b**) Distribution of deviation from target for each fEPSP amplitude value. Dotted lines indicate mean deviation for 25 ms (orange) and 50 ms flashes (blue). (**c**) The fEPSP amplitude in the time period before each flash (25 or 50 ms) demonstrates the dynamics of the response with greater time resolution.

The asymmetrical dynamics of the CPA-mediated effects on neurotransmission (fast activation, slow recovery) provide an excellent starting point for feedback-controlled light-triggered delivery of CPA. In the next set of experiments, we demonstrated the proof of principle. The goal of the real-time closed-loop was to reduce neurotransmission to a predefined target level, using the fEPSP amplitude as the control variable. During a 15 min period, baseline amplitude was determined and the target was set to 50% of this value. A representative experiment is depicted in Figure 2a. Whenever the amplitude exceeded the desired target value, it triggered a 25 ms light flash that released CPA, which reduced the fEPSP amplitude, thus maintaining a level close to the target value (Figure 2b,c). Next, we changed the duration of the light flash to 50 ms, which increased the amount of released CPA. Finally, the loop was disconnected, and the fEPSP amplitude was allowed to recover towards baseline. Under simplified assumptions, we calculated that each 25 ms light flash in this experiment led to a rise in local CPA concentration of 7 nM.

All slices (*n* = 6) showed a similar pattern, with a mean estimated CPA concentration increase of 9.6 ± 1.5 nM per 25 ms light flash and a washout time constant of 5.1 ± 1.4 min. The deviation of the fEPSP amplitude from the target in the closed-loop situation was close to the intrinsic variation during baseline. Doubling the flash duration to 50 ms released approximately twice as much CPA per flash and increased the mean time interval between flashes by a factor slightly larger than two (2.4 ± 0.2). It also significantly increased the mean deviation from the target (0.005 ± 0.001 mV versus 0.01 ± 0.002 mV for 25 and 50 ms flashes, respectively; Wilcoxon matched-pairs signed rank test, *p* < 0.05). Using the exact same illumination pattern of 25 ms LED flashes as obtained in Figure 2a, we demonstrated that, in the absence of cCPA, the light flashes had no effect on fEPSP amplitude (*n* = 3), neither at a large time scale (30 min, Supplementary Figure S4a) nor when synchronized to the single LED flashes (Supplementary Figure S4b).

These experiments demonstrate the feasibility of a closed-loop concept, which has been theorized previously [25,26], but this study is the first experimental implementation. The sample interval of 10 s cannot be reduced, but it proved sufficient for stable control of the relatively slow processes involved. At least three aspects are open for improvement: (1) there is sufficient time available for more advanced calculations with our control variable—for example, by including additional monitoring channels that could reveal relevant aspects of the network response; (2) extensions with adaptive modulation of light flash duration and higher-order control can be of help in improving the dynamic response (such as during large transients) and/or optimize the final error around the target; (3) in our slice model the response to CPA was relatively uniform (Figure 1b), but more focal application of light to fine-tune responses is another interesting path to investigate.

In conclusion, a novel coumarin-based photocaged A_1_R agonist N^6^-cyclopentyladenosine was synthesized and proved to be a useful tool for photopharmacological manipulation of neurotransmission. The asymmetrical dynamics of CPA-induced modulation appear to be well-suited for closed-loop control in neuronal circuits with unprecedented precision. Although many technical and practical difficulties still have to be overcome, this approach is expected to open up a large realm of applications in brain diseases associated with neuronal hyperexcitability, such as epilepsy.

## 3. Materials and Methods

### 3.1. Synthesis of Caged CPA Compound

Reagents and solvents of analytical grade were obtained from commercial vendors and used as received, without further purification. Moisture-sensitive reactions were conducted under a protective nitrogen atmosphere. Reactions were performed at ambient temperature, unless specifically mentioned. Analytical thin layer chromatography (TLC) was performed on precoated Macherey-Nagel^®^ F254 aluminum plates (Macherey-Nagel, Düren, Germany) that were developed by UV visualization, followed by staining with basic aq. KMnO4. Column chromatography was performed using an automated Reveleris X2 (Grace/Büchi, Flawil, Switzerland) Flash unit system with pre-packed silica columns. Exact mass measurements were recorded on a Waters LCT Premier XE™ (Waters, Zellik, Belgium) Time of Flight (ToF) mass spectrometer equipped with a standard electrospray (ESI) and modular Lockspray™ interface (Waters, Zellik, Belgium). Samples were infused as a solution of MeCN/water (1:1) + 0.1% formic acid mixture at a flow rate of 100 µL/min. NMR spectra were recorded on a Varian Mercury 300 MHz spectrometer (Palo Alto, CA, USA). Chemical shifts (δ) are reported in ppm with spectra referenced to the residual solvent peak. Coupling constants are given in Hz. Reaction monitoring and purity assessment was performed with an analytical LC/MS system (Waters AutoPurification system (Waters, Zellik, Belgium), equipped with ACQUITY QDa (mass; 100–1000 amu)) and a 2998 Photodiode Array (220–400 nm)). A Waters Cortecs^®^ (Waters, Zellik, Belgium) C18 (2.7 µm, 100 × 4.6 mm) column was employed with a gradient system of HCOOH in H_2_O (0.2%, *v*/*v*)/MeCN at a flow rate of 1.44 mL/min, 95:05 to 00:100 in 6.5 min, or a gradient of H_2_O/MeCN of 100:0 to 00:100 in 6.5 min at a flow rate of 1.44 mL/min. All obtained final compounds had purity >95%, as assayed by analytical HPLC (UV), unless otherwise specifically mentioned.

Adenosine (2.7 g, 10 mmol, 1 eq.) was suspended in DMF (50 mL, 5 mL/mmol SM) and imidazole (5.5 g, 80 mmol, 8 eq.) was added under a N2-atmosphere. Then, TBSCl (6.0 g, 40 mmol, 4 eq.) was added and the mixture stirred at ambient temperature overnight. Water was added. After stirring at ambient temperature for ~10 min, the mixture was transferred into EA/water. The layers were separated, and the organic layer was washed sequentially with sat. aq. NH_4_Cl and sat. aq. NaHCO_3_ solution and dried over Na_2_SO_4_. The filtrate was evaporated to dryness and purified by column chromatography 5→30% EA/TOL, which gave FH10716, **4** (5.5 g, 9.0 mmol) as a colorless oil that solidified upon standing. Yield = 90%. Spectra data match those reported in literature [27].

Procedure adapted from Nadler et al. [28]. 7-(diethylamino)-4-(hydroxymethyl)-2H-chromen-2-one (0.37 g, 1.5 mmol, 2 eq.) was dissolved in anhydrous THF (15 mL, 10 mL/mmol SM) under a N_2_-atmosphere. Then, DIPEA (0.39 mL, 2.3 mmol, 3 eq.) was added and the resulting solution was cooled in an ice-water bath at 0 °C. After stirring for ~5–10 min, phosgene solution (15% *w*/*v* in toluene, 2.4 mL, 3.6 mmol, 4.8 eq.) was added dropwise with a syringe pump (0.25 mL/min). After complete addition, the mixture was stirred at 0 °C for three hours. Then, it was poured onto EA/water (1/1; 100 mL), the layers were separated, and the organic layer was dried over Na_2_SO_4_, filtered and evaporated until dryness. The residue (7-(diethylamino)-2-oxo-2H-chromen-4-yl)methyl carbonochloridate (**3**) was further dried under high vacuum conditions for ~1 h and used as such. 

NMR data for compound **3**: ^1^H NMR (300 MHz, CDCl_3_) δ: 1.21 (t, *J* = 7.2 Hz, 6H, NCH_2_CH_3_), 3.42 (q, *J* = 7.2 Hz, 4H, NCH_2_CH_3_), 5.39 (d, *J* = 1.2 Hz, 2H, OCH_2_), 6.16 (t, *J* = 1.2 Hz, H-3_coum_), 6.55 (d, *J* = 2.6 Hz, 1H, H-8_coum_), 6.63 (dd, *J* = 8.9, 2.5 Hz, 1H, H-6_coum_), 7.25 (d, *J* = 9.1 Hz, 1H, H-5_coum_).

FH10716, **4** (0.46 g, 0.75 mmol, 1 eq.) was dissolved in DCM (20 mL, 40 mL/mmol SM) and pyridine (0.091 mL, 1.1 mmol, 1.5 eq.) was added. The resulting solution was cooled in a water/ice bath to 0 °C and stirred for ~5 min, after which a solution of the previously prepared (7-(diethylamino)-2-oxo-2H-chromen-4-yl)methyl carbonochloridate **3** (in DCM (10 mL)) was added dropwise with a syringe pump (0.5 mL/min). After complete addition, the solution was allowed to warm to ambient temperature and stirred for 1 h. Then, the solution was poured into DCM/water, the layers were separated and the organic layer was dried over Na_2_SO_4_, filtered and evaporated until dryness. The residue was purified by column chromatography (5→50% EA/PE) to give FH10719, **5** (0.45 g, 0.51 mmol) as a yellow foam with 68% yield. ^1^H NMR (300 MHz, CDCl_3_) δ: −0.24 (s, 3H, CH_3_), −0.04 (s, 3H, CH_3_), 0.09 (s, 3H, CH_3_), 0.10 (s, 3H, CH_3_), 0.13 (s, 3H, CH_3_), 0.15 (s, 3H, CH_3_), 0.79 (s, 9H, tBu), 0.93 (s, 9H, tBu), 0.95 (s, 9H, tBu), 1.21 (t, *J* = 7.2 Hz, 6H, NCH_2_CH_3_), 3.42 (q, *J* = 7.5 Hz, 4H, NCH_2_CH_3_), 3.80 (dd, *J* = 11.4, 2.7 Hz, 1H, H-5″), 4.03 (dd, *J* = 11.4, 3.9 Hz, 1H, H-5′), 4.13–4.17 (m, 1H, H-4′), 4.30 (t, *J* = 4.2 Hz, 1H, H-3′), 4.63 (t, *J* = 4.8 Hz, 1H, H-2′), 5.35–5.45 (m, 2H, OCH_2_), 6.09 (d, *J* = 4.8 Hz, 1H, H-1′), 6.28 (s, 1H, H-3_coumarin_), 6.56 (d, *J* = 2.1 Hz, H-8_coumarin_), 6.64 (dd, *J* = 9.3, 1.8 Hz, 1H, H-6_coumarin_), 7.38 (d, *J* = 9.0 Hz, 1H, H-5_coumarin_), 8.42 (s, 1H, H-8), 8.56 (br. s, 1H, NH), 8.78 (s, 1H, H-2). HRMS (ESI): calculated for C_43_H_71_N_6_O_8_Si_3_ ([M + H]^+^): 883.4636, found: 883.4669.

Procedure adapted from Fletcher et al. [29]. FH10719, **5** (0.27 g, 0.30 mmol, 1 eq.) and triphenylphosphine (0.12 g, 0.45 mmol, 1.5 eq.) were dissolved in anhydrous THF (4.2 mL, 14 mL/mmol SM), and cyclopentanol (0.041 mL, 0.45 mmol, 1.5 eq.) was added under a N_2_-atmosphere. The mixture was heated to 45 °C and, after 2 min at that temperature, DIAD (0.090 mL, 0.45 mmol, 1.5 eq.) was added dropwise. After complete addition, the mixture was stirred at 45 °C for 30 min, after which TLC analysis (25% EA/PE) showed full consumption of the starting material. The mixture was cooled to ambient temperature and evaporated till dryness. The residue was purified by column chromatography (5→30% EA/PE) to give FH10721, **6** (0.23 g, 0.24 mmol) as a slight yellow foam in 79% yield. ^1^H NMR (300 MHz, CDCl_3_) δ: −0.37 (s, 3H, CH_3_), −0.09 (s, 3H, CH_3_), 0.11 (s, 9H, CH_3_), 0.12 (s, 3H, CH_3_), 0.73 (s, 9H, tBu), 0.93 (s, 9H, tBu), 0.94 (s, 9H, tBu), 1.19 (t, *J* = 6.9 Hz, 6H, NCH_2_CH_3_), 1.46–1.60 (m, 2H, CH_2_), 1.61–1.71 (m, 2H, CH_2_), 1.79–1.92 (m, 2H, CH_2_), 1.97–2.09 (m, 2H, CH_2_), 3.39 (q, *J* = 7.2 Hz, 4H, NCH_2_CH_3_), 3.80 (dd, *J* = 11.7, 3.0 Hz, 1H, H-5″), 4.01 (dd, *J* = 11.4, 4.2 Hz, 1H, H-5′), 4.13–4.16 (m, 1H, H-4′), 4.31–4.33 (m, 1H, H-3′), 4.71 (dd, *J* = 5.7, 4.5 Hz, 1H, H-2′), 4.78 (quint., *J* = 8.7 Hz, 1H, CH_cyclopent_), 5.27 (d, *J* = 0.9 Hz, 2H, OCH_2_), 6.00 (s, 1H, H-3_coumarin_), 6.11 (d, *J* = 6.0 Hz, 1H, H-1′), 6.52 (d, *J* = 2.1 Hz, 1H, H-8_coumarin_), 6.59 (d, *J* = 8.4 Hz, 1H, H-6_coumarin_), 7.20 (d, *J* = 9.0 Hz, 1H, H-5_coumarin_), 8.37 (s, 1H, H-8), 8.84 (s, 1H, H-2). HRMS (ESI): calculated for C_48_H_79_N_6_O_8_Si_3_ ([M+H]^+^): 951.5262, found: 951.5298.

FH10721, **6** (0.23 g, 0.23 mmol, 1 eq.) was dissolved in MeOH. Next, NH_4_F (0.17 g, 3.7 mmol, 20 eq.) was added and the resulting solution heated at 50 °C for 2 days. Then, the solvent was removed in vacuo and the residue re-dissolved in MeOH and pre-adsorbed onto Celite^®^. Purification by column chromatography 1→7.5% MeOH/DCM gave FH12724, **2** (0.048 g, 0.079 mmol) as a slight yellow solid with 34% yield. ^1^H NMR (300 MHz, DMSO-d_6_) δ: 1.10 (t, *J* = 6.9 Hz, 6H, NCH_2_CH_3_), 1.41–1.59 (m, 4H, CH_2,cyclopentyl_), 1.70–1.82 (m, 2H, CH_2,cyclopentyl_), 1.91–1.99 (m, 2H, CH_2,cyclopentyl_), 3.41 (dd, *J* = 6.9 Hz, 4H, NCH_2_CH_3_), 3.58 (ddd, *J* = 12.0, 6.0, 4.2 Hz, 1H, H-5″), 3.69 (ddd, *J* = 12.0, 5.1, 4.5 Hz, 1H, H-5′), 3.99 (q, *J* = 3.9 Hz, 1H, H-4′), 4.17–4.22 (m, 1H, H-3′), 4.61–4.72 (m, 2H, H-2′, CH_cylopentyl_), 5.08 (t, *J* = 5.7 Hz, 1H, OH-5′), 5.26 (d, *J* = 5.1 Hz, 1H, OH-3′), 5.28 (s, 2H, OCH_2_), 5.55 (d, *J* = 6.0 Hz, 1H, OH-2′), 5.88 (s, 1H, H-3_coumarin_), 6.06 (d, *J* = 5.7 Hz, 1H, H-1′), 6.49 (d, *J* = 2.7 Hz, 1H, H-8_coumarin_), 6.60 (dd, *J* = 9.0, 2.7 Hz, 1H, H-6_coumarin_), 7.27 (d, *J* = 8.7 Hz, 1H, H-5_coumarin_), 8.88 (s, 1H, H-8), 8.90 (s, 1H, H-2). ^13^C NMR (75 MHz, DMSO-d_6_) δ: 12.3 (2C, NCH_2_CH_3_), 23.5 (2C, CH_2,cyclopentyl_), 29.7 (2C, CH_2,cyclopentyl_), 44.0 (2C, NCH_2_CH_3_), 59.9 (CH_cylopentyl_), 61.2 (C-5′), 62.7 (OCH_2_), 70.3 (C-3′), 73.7 (C-2′), 85.8 (C-4′), 87.7 (C-1′), 96.7 (C-8_coumarin_), 104.5 (C-3_coumarin_), 105.0 (C-4a_coumarin_), 108.5 (C-6_coumarin_), 125.2 (C-5_coumarin_), 130.0, 145.2, 150.4, 150.9, 151.2, 151.9, 152.9, 153.1, 155.6 (C-8a_coumarin_), 160.6 (C-2_coumarin_). HRMS (ESI): calculated for C_30_H_37_N_6_O_8_ ([M + H]^+^): 609.2667, found: 609.2673.

### 3.2. Adenosine A_1_ Receptor Competitive Binding Assay

Binding affinity of cCPA for the human adenosine A_1_ receptor (A_1_R) was measured using a radioactive competitive binding assay on membranes from human embryonic kidney (HEK)-293 cells stably expressing the human adenosine A_1_R as previously described [19]. The antagonist 8-cyclopentyl-1,3-dipropylxanthine ([^3^H]DPCPX) was used as radioligand. 

### 3.3. Acute Hippocampal Slice Recordings with a Multielectrode Array

#### 3.3.1. Slice Preparation

Twenty-eight male Wistar rats (4–7 weeks old; Janvier Labs, France) were used; they were housed with a 12/12 h light cycle and received food and water ad libitum. All experimental procedures were approved by the Animal Experimental Ethical Committee of Ghent University (ECD19/29). Animals were killed by decapitation under isoflurane anaesthesia. The brain was quickly removed and placed in ice-cold artificial cerebrospinal fluid (aCSF) of the following composition (mM): NaCl 124, NaHCO_3_ 26, KCl 2, KH_2_PO_4_ 1.25, CaCl_2_ 2, MgSO_4_ 2 and glucose 10, saturated with carbogen (95% O_2_/5% CO_2_) (pH = 7.4). Transverse slices (350 µm) were cut from the ventral hippocampus using a VT1200S microtome (Leica Microsystems, Wetzlar, Germany) and were placed in carbogen-saturated aCSF at room temperature for at least one hour until use.

#### 3.3.2. Electrophysiological Recordings

Slices were transferred to the multielectrode array (MEA) chamber and continuously incubated with carbogen-saturated aCSF at a flow rate of 2 mL/min. All solutions were preheated to 34 °C using a heatable perfusion cannula (PH01, MultiChannel Systems (MCS), Reutlingen, Germany) at the inlet of the MEA chamber, where temperature was maintained at 31 °C. Slice positioning was ensured by a custom-made nylon grid. Field potentials were recorded with a planar 60 channel MEA (MCS, Reutlingen, Germany), containing 60 TiN-coated electrodes (30 µm diameter and 200 µm spacing), one of which was used as A common reference. Voltage signals were acquired with an MEA1060-BC high-bandwidth pre-amplifier and digitized at 25kHz with 16 bit resolution using MC_Rack software (Version 4.6.2, MCS, Reutlingen, Germany). After an equilibration period of at least 15 min, slice quality was tested by applying a monophasic negative voltage pulse (amplitude 2000 mV, duration 100 µs) to one of the electrodes located in the CA1 region near the Schaffer collaterals. The stimulation parameters (duration, amplitude and repetition rate) were controlled by a programmable stimulator (STG4002; MCS, Reutlingen, Germany). Only experiments where at least three channels in the Stratum Radiatum within the CA1 region showed a field potential amplitude larger than 200 µV were included. In all subsequent experiments, field potentials were evoked by repeated stimulation with an interval of 10 s. Optical stimulation was delivered by a 405 nm light-emitting diode (LED) (M405FP1, Thorlabs, Bergkirchen, Germany), positioned diagonally above the MEA chamber, its timing being computer-controlled. Energy output from the LED, measured with a photodiode power sensor (S120VC, Thorlabs, Bergkirchen, Germany) placed at the MEA chamber position, was set at 4.0 mW. All optical experiments were performed in the dark.

#### 3.3.3. Experimental Paradigms

Field potential amplitude resulting from Schaffer collateral stimulation of increasing voltages (500 to 3500 mV in steps of 250 mV) yielded a stimulus–response curve from which the stimulus intensity (I_50_) was determined for subsequent experiments. This was defined as the stimulus that evoked a field potential amplitude reaching 50% of its value obtained at the first stimulation intensity yielding the largest response. In this way, we monitored synaptic strength at a sensitive and comparable intensity. Slices were incubated with aCSF that contained 3 µM cCPA while monitoring the fEPSP amplitude.

After establishing a baseline for at least 15 min, the slices were exposed to a single light flash of different duration (500, 1000 or 2000 ms) that released CPA. 

As a control variable for feedback-controlled triggered light application, we selected an MEA channel that showed a relatively pure dendritic negative response, hereafter called the field excitatory postsynaptic potential (fEPSP). During the experiment, fEPSPs were evoked with a fixed stimulation amplitude (I_50_). Baseline fEPSP amplitude was estimated during a 15 min baseline recording. The desired target amplitude level was set to 50% of baseline value. Once the loop was closed, fEPSP amplitude level crossing triggered a fixed LED flash of 25 ms duration that released CPA. After 30 min, the duration of the light flash was changed to 50 ms. Control slices were exposed to the illumination train obtained in the experiment illustrated in Figure 2a, in the absence of cCPA.

#### 3.3.4. Data Analysis

Offline analysis of fEPSPs was done using custom-written MATLAB-software (MathWorks, Natick, MA, USA). The amplitude of negative fEPSPs in the Stratum Radiatum was calculated as the difference between the minimal value and the zero value determined 2 ms before the stimulus. Slices with more than 15% fEPSP amplitude variation during the last 5 min of the baseline recording period (*n* = 11) were excluded from further analysis. 

For the comparison of fEPSP amplitude changes in response to cCPA incubation and single LED flashes in different slices, amplitude was normalized to the mean value obtained in the last five minutes of baseline recording (before the perfusion switch and the light flash delivery, respectively). The induction rate of the CPA-induced amplitude reduction was estimated by a least square regression of the falling slope of the time course curve of the fEPSP amplitude during the first 30 s after the LED flash. The recovery time constant of these responses was estimated by a least square fit of a single exponential function over about 20 min, starting a few minutes after the minimum value was reached.

In the closed-loop experiment, online analysis of fEPSP amplitude level crossing was undertaken using the MC_Rack built-in software (MCS, Reutlingen, Germany). In a first approximation, two parameters determined the time course of fEPSP amplitude during the closed-loop experiment: (1) the fixed amount of CPA released per light flash, resulting in a local increase of y nM CPA and (2) the CPA wash-out time constant of λ min (if we assume constant single exponential wash-out of CPA during the whole experiment). During the initiation phase, which started immediately after closing the loop, each fEPSP amplitude value was above target level and initiated a light flash until the target level was reached, most often with an initial undershoot before finally recovering to the target value. In these proof-of-concept experiments, we set the target value to 50% of the baseline fEPSP amplitude and assumed that the CPA concentration within the slice was around the IC_50_ value for fEPSP suppression, reported to be around 30 nM [20,21,22]. The following equation describes the concentration of CPA attained at the last flash given at time T_n_ as a result of all other flashes of identical size y given at times T_i_ (1 to n) in the train, assuming single exponential removal of CPA with a time constant λ:

$${\mathrm{IC}}_{50}=\left[\mathrm{CPA}\right]=\overset{n}{\underset{i=1}{\sum }}\left(y\times \exp\frac{T_{i}-T_{n}}{\lambda }\right)$$

The initiation phase was followed by a steady-state phase where the same equation held, resulting in two equations with unknowns y and λ that could be solved. 

In the control slices, a small global trend (from 10 min before the train until the end of the train) was removed where present and fEPSP amplitudes were normalized to baseline value, determined in the five minutes before the train started. We also constructed the mean fEPSP amplitude response immediately after a single LED flash, normalized to the last amplitude value before the flash (99 flashes from three different slices).

#### 3.3.5. Statistical Analysis

Statistical analysis was performed with Prism software (Version 8.0.2, GraphPad, San Diego, CA, USA) and MATLAB (MathWorks, Natick, MA, USA). Unless indicated otherwise, data are reported as the mean ± standard error of the mean (SEM), where *n* represents the number of slices. The nonparametric Kruskal–Wallis test followed by post hoc Dunn’s test was used for multiple comparisons of the effect of light flash duration, induction rate and recovery time constant. The nonparametric Wilcoxon matched-pairs signed rank test was used to test the effect of doubling the flash duration on the mean deviation from the target. *p* < 0.05 was assumed to reject the null hypothesis.

#### 3.3.6. Chemicals

All chemicals were obtained from Sigma-Aldrich (St. Louis, MO, USA). cCPA was made up as a 3 mM stock solution in 100% dimethylsulfoxide (DMSO) and stored at −20 °C. The final perfusion solutions always contained 0.1% DMSO.

## Data Availability

The data presented in this study are available from the corresponding author on reasonable request.

## Supplementary Materials

The following supporting information can be downloaded at: https://www.mdpi.com/article/10.3390/ijms23168887/s1.
