# Supplementary material for: Ex Vivo Feedback Control of Neurotransmission Using a Photocaged Adenosine A1 Receptor Agonist
Source: Int J Mol Sci. 2022 Aug 10;23(16):8887. doi: 10.3390/ijms23168887 (PMC9408941; doi:10.3390/ijms23168887)
Supplement: Supplementary file 1 [file ijms-23-08887-s001.zip › ijms-1783848-supplementary.pdf]

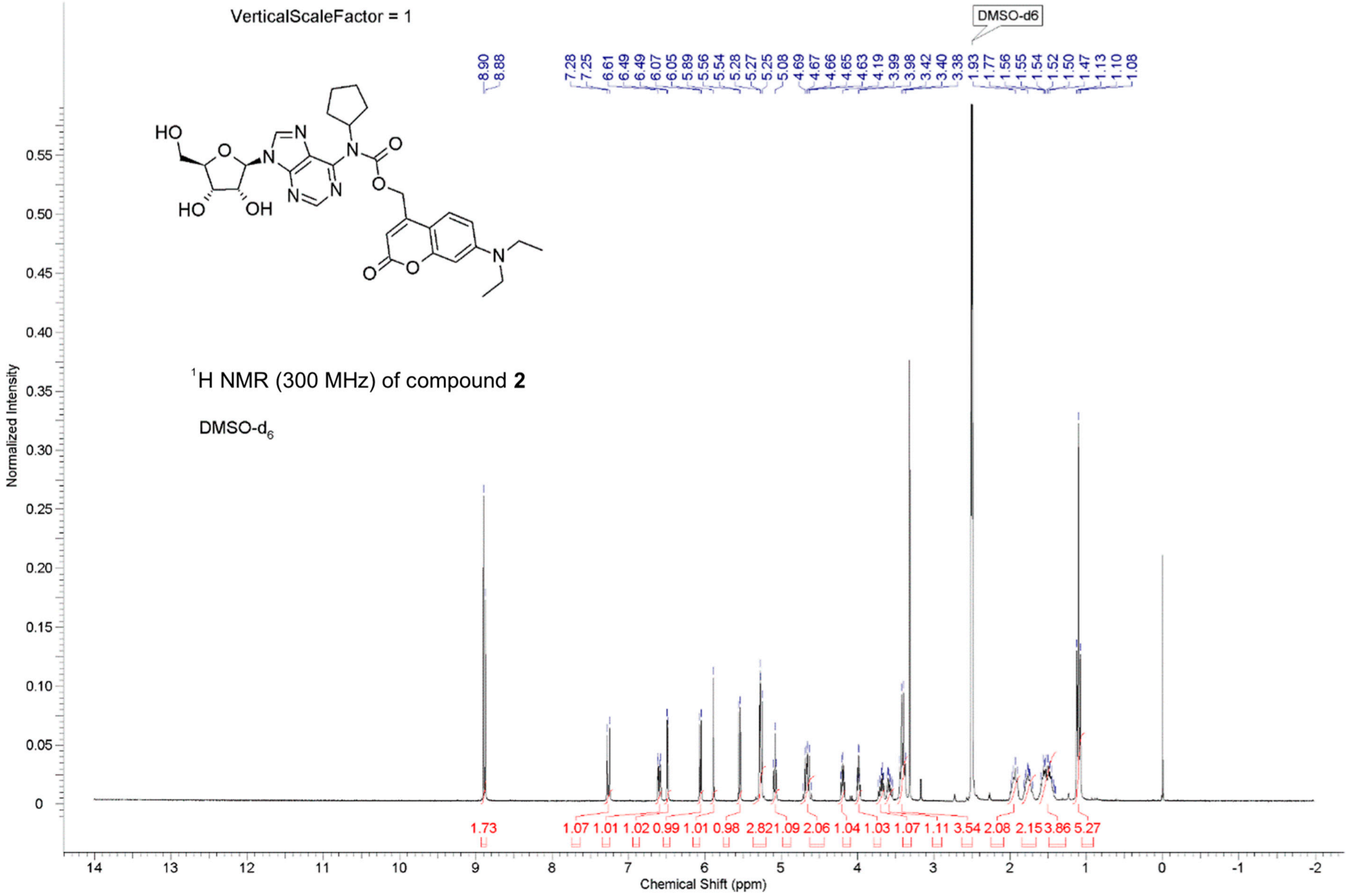
VerticalScaleFactor = 1
DMSO-d6
8.90 8.88 7.28 7.25 6.61 6.49 6.49 6.07 6.05 5.89 5.56 5.54 5.28 5.27 5.25 5.08 4.69 4.67 4.66 4.65 4.63 4.19 3.99 3.98 3.42 3.40 3.38 1.93 1.77 1.56 1.55 1.54 1.52 1.50 1.47 1.13 1.10 1.08
HO
HO
OH
N
O
^{1}H NMR (300 MHz) of compound **2**
DMSO-d_6
Normalized Intensity
1.73 1.07 1.01 1.02 0.99 1.01 0.98 2.82 1.09 2.06 1.04 1.03 1.07 1.11 3.54 2.08 2.15 3.86 5.27
Chemical Shift (ppm)

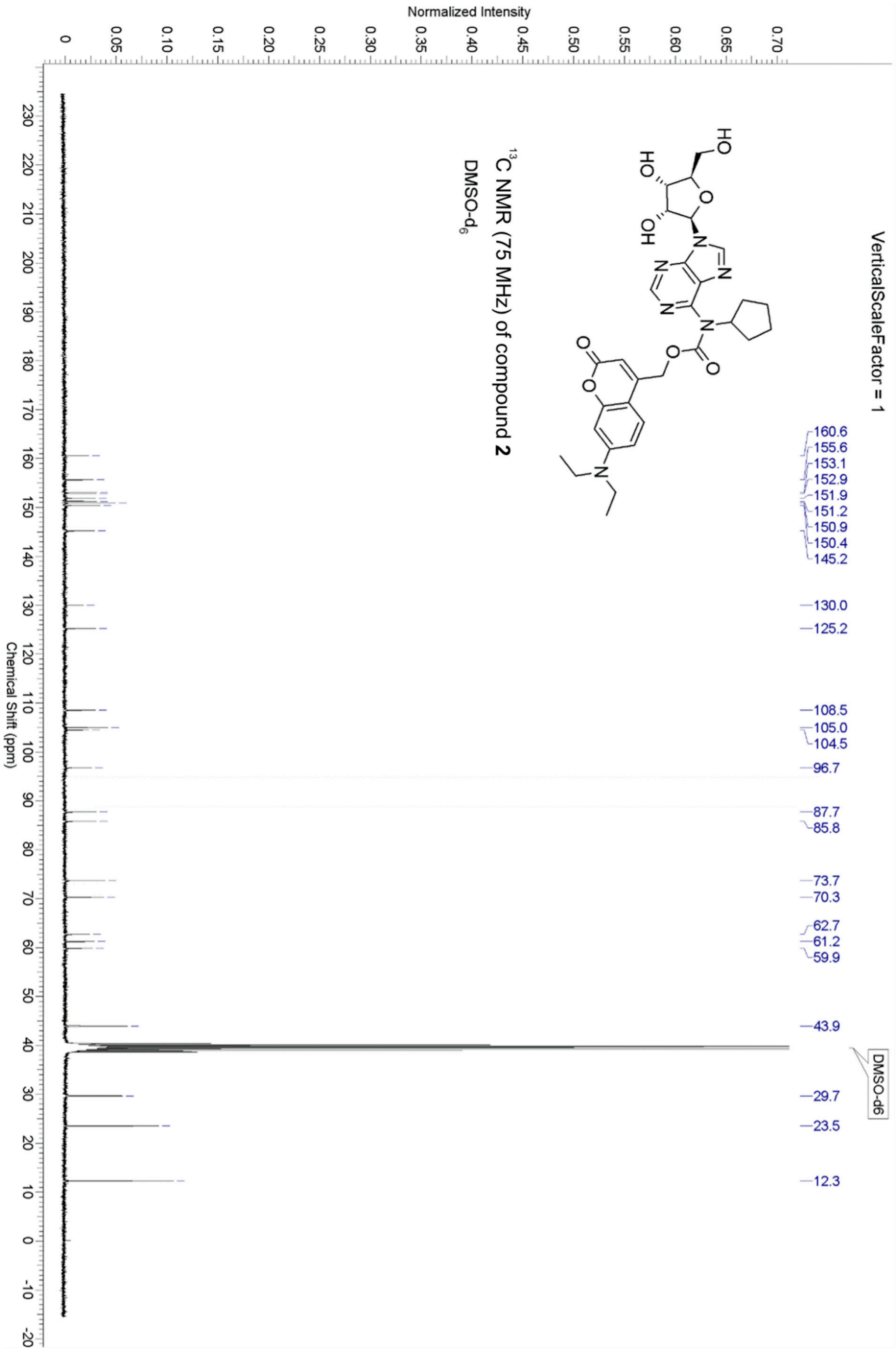

Figure S1. Supporting NMR spectra for the synthesis of cCPA.

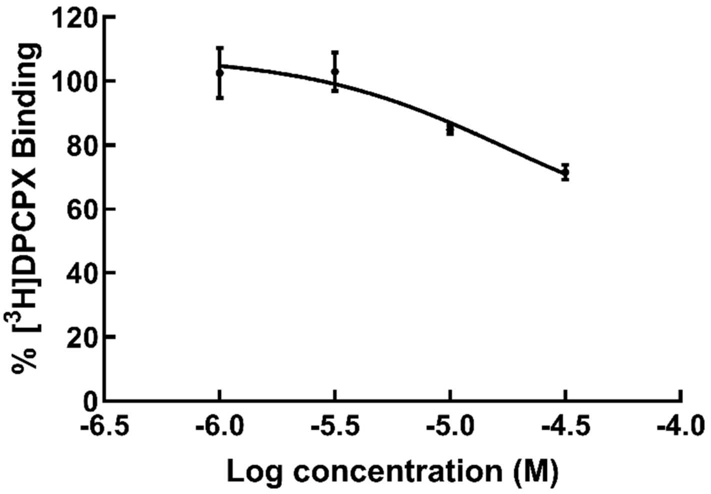

Figure S2. Displacement of [^{3}H]DPCPX binding to the A_1R by cCPA measured in HEK293 cell membranes stably expressing hA_1Rs. Data are expressed as mean ± standard error of the mean (SEM) of two experiments.

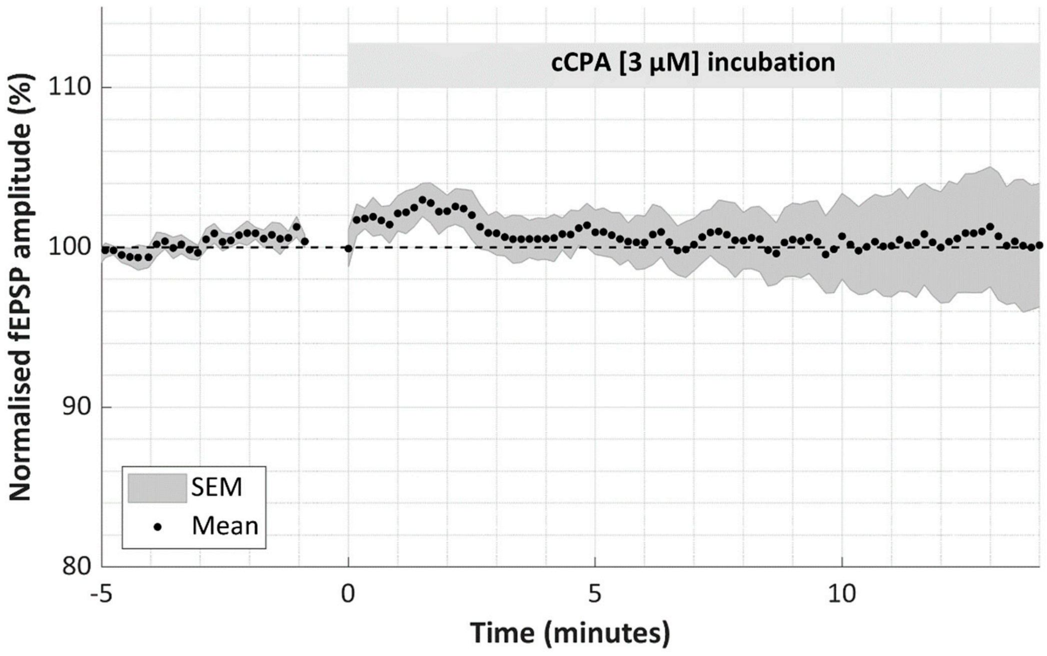

Figure S3. Normalized fEPSP amplitude as a function of time (determined once every 10 seconds) before and during incubation with cCPA [3μM], starting at zero time. Data was normalized to the mean value before incubation with cCPA and expressed as mean ± SEM (n=14). We conclude that cCPA does not affect the fEPSP amplitude.

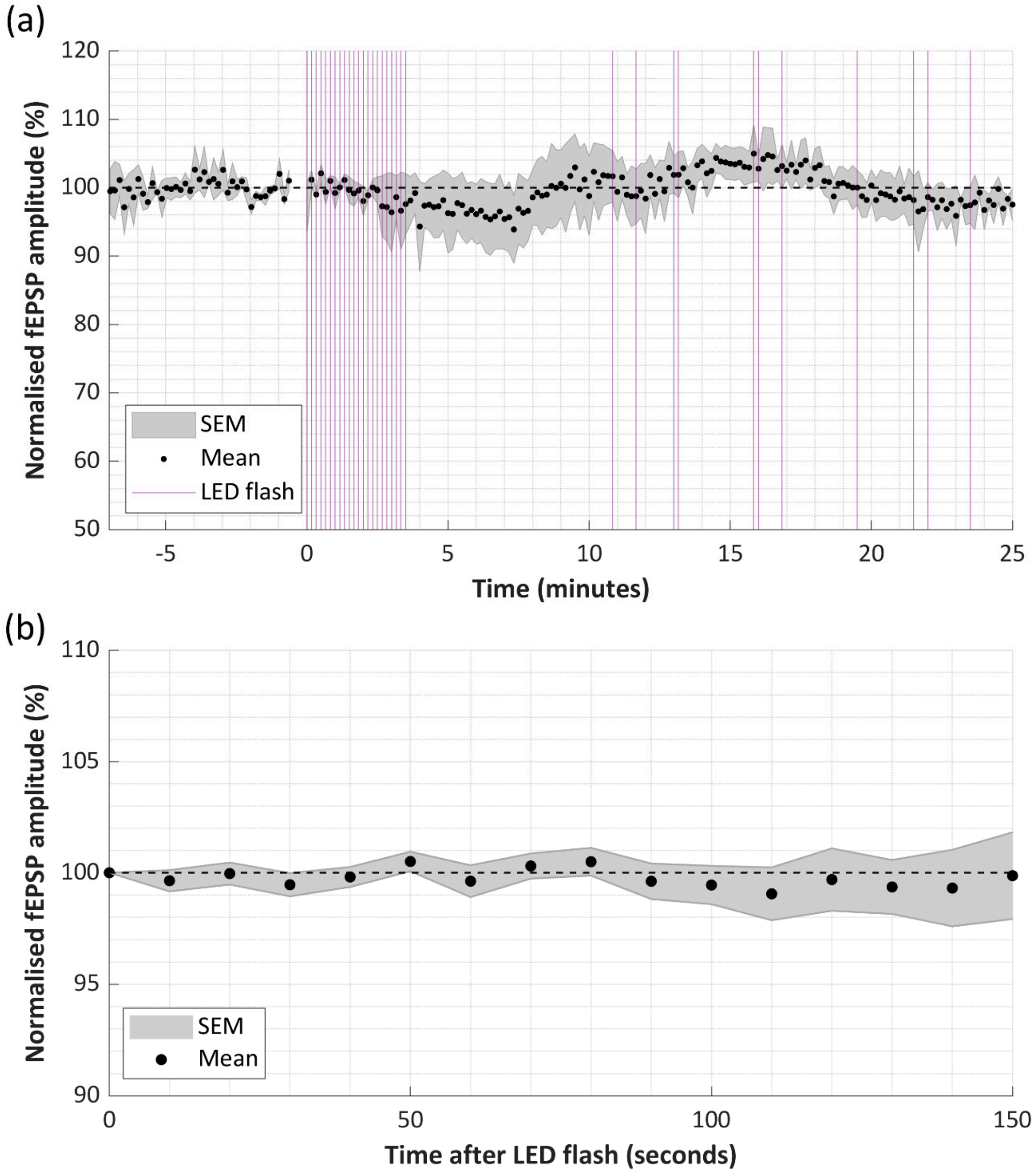

Figure S4. (**a**) Normalized fEPSP amplitude as a function of time (determined once every 10 seconds) to determine the effect of a train of 25 ms LED flashes (pattern identical to the train obtained in Figure 2a) in the absence of cCPA. Data was normalized to the mean value before the start of the light flash train. We conclude that the light flashes in the absence of cCPA do not affect the fEPSP amplitude. (**b**) Reorganized data from (**a**) to investigate the effect of a single LED flash on the fEPSP amplitude. Time course of the fEPSP amplitude immediately after each 25 ms LED flash. Data was normalized to the value before the light flash. Because the LED flash intervals differ, later time points in this curve contain less observations. Here, we also conclude that the light flashes have no effect on the fEPSP amplitude in the absence of cCPA. All data are expressed as mean ± SEM (*n*=3).
